# Soft tissue grafting at immediate implants: does it protect the buccal plate?

**DOI:** 10.1038/s41432-026-01234-y

**Published:** 2026-07-07

**Authors:** Sunkanmi Olaore, Pynadath George, Kathryn Taylor

**Affiliations:** 1 University of Lancashire, Preston, UK; 2 Kesgrave Dental Care, Ipswich, UK

**Keywords:** Dental implants, Cone-beam computed tomography

## Abstract

**A Commentary on:**

**Jung M, Tran D, Chang CC, Kim SK, Tsukiboshi Y, Min S, Ayilavarapu S, Lee CT**.

Volumetric buccal bone alterations at immediate implant sites with or without soft tissue augmentation: A 6-month assessment. J Periodontol. 2026;97:220–33. https://doi.org/10.1002/jper.11381.

**Design:**

This was a secondary analysis of cone-beam computed tomography (CBCT) and scanned model data from a registered randomised controlled trial evaluating immediate implant placement with or without soft tissue augmentation^1^. The study compared immediate implant placement with subepithelial connective tissue grafting, immediate implant placement with acellular dermal matrix, and immediate implant placement without soft tissue augmentation (control).

**Case selection:**

Forty-five patients requiring extraction of a single maxillary anterior or premolar tooth followed by immediate implant placement were included. Participants were allocated to one of three groups: immediate implant with subepithelial connective tissue graft, immediate implant with acellular dermal matrix, or immediate implant alone. In all groups, the gap between the implant and the alveolar bone was filled with bovine bone material and a collagen dressing was placed.

**Data analysis:**

CBCT scans were taken before extraction and 6 months after immediate implant placement. The scans were superimposed to assess three-dimensional volumetric and two-dimensional linear buccal bone dimensional changes. The buccal region was divided into coronal, middle and apical thirds. Volumetric bone loss was calculated in absolute and relative terms. Linear horizontal and vertical bone changes were also assessed. Measurements were carried out by a calibrated blinded examiner. Statistical analysis included ANOVA or Kruskal–Wallis tests, linear regression analysis to explore associations between clinical factors and volumetric bone change, and Pearson correlation coefficients to compare 3D and 2D measurements.

**Results:**

There was no statistically significant difference in volumetric buccal bone loss between the three groups at 6 months. The greatest loss occurred in the coronal third, with mean volumetric reductions of −14.99 ± 4.29 mm³ in the connective tissue graft group, −13.60 ± 3.97 mm³ in the acellular dermal matrix group, and −12.58 ± 4.09 mm³ in the control group (p = 0.286). Thick bone morphotype was associated with greater absolute volumetric bone loss. Correlations between 3D and 2D measurements varied between anatomical segments.

**Conclusion:**

Soft tissue augmentation using either a subepithelial connective tissue graft or acellular dermal matrix did not significantly reduce buccal bone dimensional loss at immediate implant sites after 6 months. Linear measurements may not represent fully volumetric buccal bone changes.

## GRADE Rating:



## Risk-of-bias judgement

Using the Cochrane RoB 2 framework, the main outcome of volumetric buccal bone dimensional change at 6 months was judged to have some concerns. Although the study was based on a randomised controlled trial and outcome assessment was performed by a calibrated blinded examiner with high intra-examiner reliability, volumetric buccal bone analysis was not planned in the original trial^1^. In addition, the sample size calculation was based on soft tissue contour change rather than buccal bone dimensional change, so the study may have been underpowered to detect clinically relevant differences in the bone outcome. There was no clear evidence of important deviations from intended interventions, missing outcome data, or bias in outcome measurement. The principal concern lies in the selection of the reported result. The volumetric analysis was non-prespecified and lacked a bone-specific power calculation, supporting an overall judgement of some concerns.

## Commentary

Preservation of the buccal plate remains a key challenge in immediate implant placement, particularly in the aesthetic zone. Soft tissue augmentation is used commonly to increase peri-implant mucosal thickness, support soft tissue contour and reduce the risk of recession. A related clinical assumption is that improving the soft tissue phenotype may also help preserve the underlying buccal bone. This study challenges that assumption^2^.

Neither subepithelial connective tissue grafting nor acellular dermal matrix reduced volumetric buccal bone loss compared with no augmentation, and this held for both absolute and relative measurements (coronal third *p* = 0.286 and *p* = 0.396 respectively). This is relevant clinically because both grafting approaches add treatment complexity, cost and surgical burden, with additional donor-site morbidity for autogenous connective tissue grafts. Any routine use therefore requires clear clinical justification.

An important point of interpretation, however, is that all three groups received bovine bone in the implant-to-bone gap together with a collagen dressing. Gap grafting is itself a ridge-preserving intervention, and the authors note it may compensate for the effect of a thin buccal plate. The trial therefore tests whether soft tissue augmentation adds a measurable bone benefit on top of gap grafting, rather than whether augmentation preserves bone in isolation. A shared, effective ridge-preserving step in every arm narrows the ability for augmentation to show a further effect, and this should influence how the null result is read.

A strength of the study is the use of volumetric CBCT analysis. Many implant studies rely on linear measurements from selected cross-sectional slices, which may not capture the full pattern of buccal bone remodelling^3,4^. The variable correlation between 2D and 3D measurements, ranging from weak to high across segments, supports the view that linear measurements alone may not fully represent dimensional bone change. The use of a single calibrated, blinded examiner with high intra-examiner reliability further strengthens confidence in the measurements.

The finding that a thicker bone morphotype was associated with greater absolute volumetric bone loss should not be misread as evidence that thicker plates are disadvantageous. The authors show that thicker plates accompanied wider baseline ridges, which demonstrated increased bone loss in absolute terms but not in relative (proportional) terms. This distinction is of clinical significance, and it is worth noting that the patient samples were predominantly thin-plated (mean buccal thickness approximately 0.6–0.7 mm), whereas the thicker morphotype represented a minority of sites.

These secondary associations should also be read with caution, because the regression and correlation analyses involved many comparisons at the conventional 5% significance level with no adjustment for multiple testing. The associations reported for bone morphotype, horizontal defect dimension and keratinised tissue width are therefore best regarded as exploratory and hypothesis-generating rather than confirmatory.

Even so, the findings should be interpreted cautiously. The volumetric bone analysis was a secondary, non-prespecified analysis of a trial powered for soft tissue contour change, with no sample-size calculation for the bone outcome. The absence of a statistically significant difference does not establish that augmentation has no effect on buccal bone; it may reflect insufficient power to detect a smaller but clinically relevant difference. Follow-up was limited to 6 months, capturing early remodelling but not longer-term peri-implant bone stability, recession, aesthetics or peri-implant health. A minor additional limitation is that two of the 6-month scans were acquired on a different CBCT unit, although the same voxel size and a standardised protocol were used.

For practising clinicians, the message is not that soft tissue augmentation lacks value, but that its value should be defined more precisely. It remains useful for phenotype modification, soft tissue contour support and aesthetic management, but should not be presented primarily as a method of preventing buccal bone loss^1^. Implant positioning, atraumatic extraction, buccal plate integrity, gap grafting, emergence profile design and long-term maintenance remain central to tissue stability around immediate implants.

Further research should include adequately powered randomised trials in which volumetric buccal bone change is a pre-specified primary outcome, with longer follow-up to determine whether early volumetric changes translate into meaningful differences in recession, aesthetics, peri-implant health and implant survival.

Practice point

- Soft tissue augmentation at immediate implant placement has not been shown to prevent buccal bone loss and should be selected for soft tissue phenotype, contour and aesthetic indications rather than bone preservation. As current evidence from analyses not powered for bone outcomes, a small protective effect cannot yet be excluded.
